# Circ-CCDC66 upregulates REXO1 expression to aggravate cervical cancer progression via restraining miR-452-5p

**DOI:** 10.1186/s12935-020-01732-8

**Published:** 2021-01-06

**Authors:** Yan Zhang, Xing Li, Jun Zhang, Lin Mao

**Affiliations:** Department of Obstetrics and Gynecology, Renmin Hospital of Wuhan University, Wuhan, China

**Keywords:** Circular RNA, Circ-CCDC66, miR-452-5p, REXO1, Cervical cancer

## Abstract

**Background:**

Cervical cancer is one most common cancer types among females over the world. While its underlying mechanisms remain unclear. Circ-CCDC66 has been revealed to participate in multiple biological functions, and contribute to various diseases’ progression. In the current study, we aimed to demonstrate the role of circ-CCDC66 in cervical cancer progression.

**Methods:**

Real-time quantitative PCR (RT-qPCR) was conducted to measure the expression of circ-CCDC66, miR-452-5p, and REXO1 mRNA. Cell fractionation assay and RNA fluorescence in situ hybridization (FISH) were performed to locate circ-CCDC66 in cells. Cell account kit 8 (CCK-8) was used to detect cell proliferation ability. Transwell assay was applied to evaluate cell migration or invasion ability. Bioinformatics analysis, biotinylated RNA pull-down, RNA immunoprecipitation, and dual-luciferase reporter assays were conducted to assess the association between miR-452 and circ-CCDC66 or REXO1. Western blot was applied to measure the protein expression of REXO1. The animal tumor model was used to assess the effect of circ-CCDC66 in vivo.

**Results:**

The expression of circ-CCDC66 was upregulated in cervical cancer tumor tissues in comparison with normal tissues, and correlated with later tumor stage and larger tumor size. Downregulated circ-CCDC66 inhibited cervical cancer cell proliferation, migration, and invasion. Circ-CCDC66 was an efficient molecular sponge for miR-452-5p, and negatively regulated miR-452-5p expression. MiR-452-5p directly targeted to REXO1. Circ-CCDC66 regulated REXO1 expression to modulate cervical cancer progression via miR-452-5p. Moreover, downregulated circ-CCDC66 was found to suppress tumor growth in vivo*.*

**Conclusion:**

Our results demonstrated the role of circ-CCDC66/miR-452-5p/REXO1 axis in cervical cancer progression, we might provide novel therapeutic targets for cervical cancer clinical intervention.

## Highlights

1. Circ-CCDC66 was upregulated in cervical cancer tumor tissues.
2. Downregulated circ-CCDC66 inhibited cervical cancer cell proliferation, migration, and invasion.
3. Circ-CCDC66 upregulated REXO1 expression to modulate cervical cancer progression via sponging miR-452-5p.
4. Downregulated Circ-CCDC66 inhibited cervical cancer cell growth in vivo.

## Background

Cervical cancer is one most common malignancy types of gynecological tumors, and a primary reason caused cancer-related death to female worldwide [1]. In china, cervical cancer is the sixth most common cancer type, which causes more than 34,000 death in 2015 [2]. Despite the application of vaccine has been widespread, and the diagnosis and intervention of cervical cancer have made great improvement, 25% of patients diagnosed with cervical cancer still fail to recover due to the high recurrence and distance metastasis [3, 4]. Cervical cancer has brought huge health burden for world health care system. It is imperative to find novel targets for cervical cancer treatment.

Circular RNA (circRNA) is one type of non-coding RNAs, which is characterized by its covalently closed loop structure [5]. Due to its stable, conserve, and spatio-temporal specificity futures, circRNAs are abundantly expressed in human tissues, and involved in various cellular progression [6, 7]. In the past decades, the functions of circRNAs in multiple biological progressions have been deeply studied, including cervical cancer [8–11]. Gao et al. revealed that has_circ‐0018289 mediates cervical cancer cell proliferation, invasion, and migration via acting as a molecular sponge for miR-497 [12], Song T et al. elucidated the role of hsa_circRNA_101996 in cervical cancer development through regulating miR-8075/TPX2 axis [13], Tang Q et al. demonstrated that hsa_circ_0000515 mediates cervical cancer progression via miR-326/ELK1 pathway [14]. Accumulating evidence suggest that circRNAs play essential role in the initiation or progression of cervical cancer. CircRNA circ-CCDC66 (hsa_circ_0001313) derives from chr3:56626997–56628056 and consists about 460 nts [15]. Circ-CCDC66 was found to participate in Hirschsprung's disease, Colon cancer, Gastric cancer, and Abdominal Aortic Aneurysm development via regulating multiple cellular progressions [16–19]. However, the role circ-CCDC66 in cervical cancer development is still uncovered.

In this study, we aimed to investigate the role of circ-CCDC66 in cervical cancer progression. Firstly, we measured the expression of circ-CCDC66 in thirty-six pairs of cervical cancer human samples. Next, we assessed the biological functions of circ-CCDC66 in cervical cancer cells. Subsequently, by using bioinformatic analysis, luciferase reporter assays, circ-CCDC66 was found to upregulate REXO1 expression via sponging miR-452-5p. Collectively, our study demonstrated the role of circ-CCDC66/miR-452-5p/REXO1 pathway in cervical cancer progression. We might provide new insights for cervical cancer basic research and novel therapeutic targets for clinical management.

## Materials and methods

### Clinical human samples

Thirty-six pairs human samples were collected from cervical cancer patients who underwent surgery in the department of obstetrics and gynecology, Renmin Hospital of Wuhan University from 2018 to 2019. The cervical cancer patients who received chemotherapy, immunotherapy, or radiotherapy were not enrolled. All specimens (2 cm away from the tumor tissues) were exanimated and approved by two pathologists, respectively. All samples were immediately froze and stored at −80 °C after operation. All patients or their families were informed the approach of sample collection, and informed consent were obtained. All experiments in this study were approved by the Ethics Committee of the Renmin Hospital of Wuhan University.

### Cell culture and transfection

All cervical cancer cell lines C33A, HT-3, HeLa, SiHa, normal cervical cell H8, and HEK-293 T cells were commercially obtained from American Type Culture Collection (ATCC, USA). Cells were maintained in Dulbecco's Modified Eagle Medium (DMEM, Gibco, USA) with 10% fetal bovine serum (FBS, Gibco, USA) and 1% streptomycin double-antibody at 37 °C with 5% CO_2_. Sh-circCCDC66 and its normal control shRNA were constructed by GeneChem (Shanghai, China). Circ-CCDC66 or REOX1 sequences were subjected to pcDNA3.1 vector to generate circCCDC66 or REXO1 overexpression vectors by Genepharma (Suzhou, China). MiR-452-5p mimic and its normal control mimic were constructed and commercially procured from Sangon Biotech (Shanghai, China). Lipofectamine 3000 (Invitrogen, USA) were applied to carry out all transfections.

### Animal experiment

The 8-week old nude mice (28 for study) were purchased from Vital River Inc. (Beijing, China). The ethics committee of the Renmin Hospital of Wuhan University approved animal experiments in this study. HeLa cells were stably transfected with Sh-circCCDC66 and Sh-NC. Then, about 1 × 10^7^ treated cells (per tumor) were subcutaneously injected into nude mice. After 28 days, mice were sacrificed, and tumors were harvested. The animal experiments were followed with the ethical standard of Helsinki Declaration of 1975 (1983 reversion).

### Real-time qPCR

Total RNAs from cervical cancer human samples and cells were collected by Trizol kit (Invitrogen, USA). A PrimerScript RT Reagent kit (TaKaRa, Japan) was used to reversely transcribed RNA into cDNA. The amplification was conducted using SYBR Green dye. GPADH was used as an internal reference. The reliability of PCR results was confirmed by the comparative CT(2^−ΔΔCt^) method.

The information about primers used in study is showed in the supplementary Table 1.

**Table 1:** Information of primers used for real-time polymerase chain reaction

Primer	Sequence (5′ to 3′)
CCDC66	ACCTACAACCGGAAGCCAG
	AGCAGTACTGTTTCCTGATGC
miR-452-5p	AAGAGGGCATGGAAACACTG
	ACTCACCCCATTCTTCAAGG
REXO1	GTATGACCCTCTGTCCAACTTCTCT
	CATCATCATCTGAGTCTGAGAACCT
GAPDH	GGTCTCCTCTGACTTCAACA
	GTGAGGGTCTCTCTCTTCCT
U6	GCTTCGGCAGCACATATACTAAAAT
	CGCTTCACGAATTTGCGTGTCAT

### Western blot

Total proteins from cervical cancer tissues and cells were collected using a RIPA lysis buffer (Beyotime, Shanghai, China). Proteins were separated by 10% SDS-PAGE, and then transferred to PVDF membranes (Millipore, USA). Membranes were incubated with primary antibodies over night at 4 °C, and then with secondary antibodies at room temperature for 2 h. Protein bands were visualized by the ECL Western Blotting Detection Kit (PA, USA). Primary antibodies as following: AGO2 (1:1000, CST, 2897S), IgG (0.5 µg/ml, Abcam, ab190475), REXO1 (0.2 µg/ml, Abcam, #ab243536), GAPDH (1:2000, Abcam, #ab8245).

### RNA fluorescence in situ hybridization (FISH)

Fluorescent In Situ Hybridization Kit (RiboBio, China) was used to carry out RNA FISH assay according to manufacturer’s instruction. Cy3-labeled circ-CCDC66 probes were procured from GeneChem (Shanghai, China). 4% paraformaldehyde was adopted to fix cells. Subsequently, cells were treated by permeabilization with 0.5% Triton X-100, then subjected to hybridization solution. After that, treated cells were incubated with Cy3-labeled circ-CCDC66 probes overnight. The results were detected by Fluorescent In Situ Hybridization Kit and visualized with a confocal microscopy.

### Cell proliferation experiment

Cell proliferation ability was detected by cell account kit 8 (CCK-8, Dojindo, Japan) according to a previous study [20]. Collectively, HeLa and SiHa cells (1 × 10^4^ per well) were seeded into a 96-well plate and housed for 3 days. CCK‐8 solution (10 μl) was added into well, two hours before detection. The absorbance at a wavelength of 450 nm was recorded, and repeated three times.

### Cell migration and invasion experiment

Transwell chamber with 8.0 μm pores (Corning, USA) was used to detect cell migration and invasion ability. For migration, the upper chambers were seeded with cells (1 × 10^4^ per well) with DMED, and the lower chambers were added with DMED with 10% FBS. After 2 days, the cells on the lower surface were fixed with 4% paraformaldehyde, and visualized under a microscope. For invasion, Matrigel (BD, USA) was used to cover upper chambers, cells were incubated on the upper chambers with Matrigel and DMEM, lower chambers were filled with DMEM with 10% FBS. After 2 days, cells attached on lower surface were fixed with 4% paraformaldehyde, and visualized under a microscope. Experiments were carried out three times.

### RNA pull-down

The biotinylated RNA pull-down assay was carried out followed by previous studies [21, 22]. Biotinylated CCDC66 and miR-452-5p probes were synthesized and commercially obtained from Sangon Biotech (Shanghai, China). Briefly probe-coated beads were generated using C-1 magnetic beads (Life Technologies, Carlsbad, CA, USA). Cell lysates were collected using lysis buffer (Sigma, Germany), subsequently, incubated with C-1 magnetic beads at 4 °C for 120 min. Then, elution buffer (Sigma-Aldrich) was used to elute precipitated RNAs from beads. RNA bands were analyzed by qRT-PCR.

### AGO2-RIP

RNA immunoprecipitation assays using anti-AGO2 and anti-IgG antibodies were performed using a Magna RIP™ RNA-Binding Protein Immunoprecipitation Kit (Millipore, Bedford, MA, USA) according to manufacturer’s protocol. Then, RNA complexes were subjected to qRT-PCR assay. Experiments were repeated three times.

### Dual luciferase assay

The sequences of CCDC66 and REXO1 3′UTR containing wide-type or mutant-type miR-452-5p binding sites were subjected to pGL3-Basic luciferase vector (Promega, USA). Then, vectors were transfected into 293 T and HeLa cells with pRL-TK vector (Promega), miR-452-5p mimic and its normal control mimic. After 2 days, firefly activity was detected in comparison with the Renilla control by a Dual-Luciferase Reporter assay system (Promega).

### Statistical analysis

All data from experiment were presented as mean ± Standard Deviation (SD). SPSS 17.0 software (SPSS, USA) was used to carry out results data. All experiments were repeated at least three times. The differences between two groups were calculated by student t-test. The differences between multiple groups were analyzed by Analysis of Variance (ANOVA). The statistical correlation between miR-452-5p and circ-CCDC66 or REXO1 were calculated by spearman analysis. p < 0.05 was considered statistically significant.

## Results

### Circ-CCDC66 is upregulated in cervical cancer tissues

**Fig. 1:**
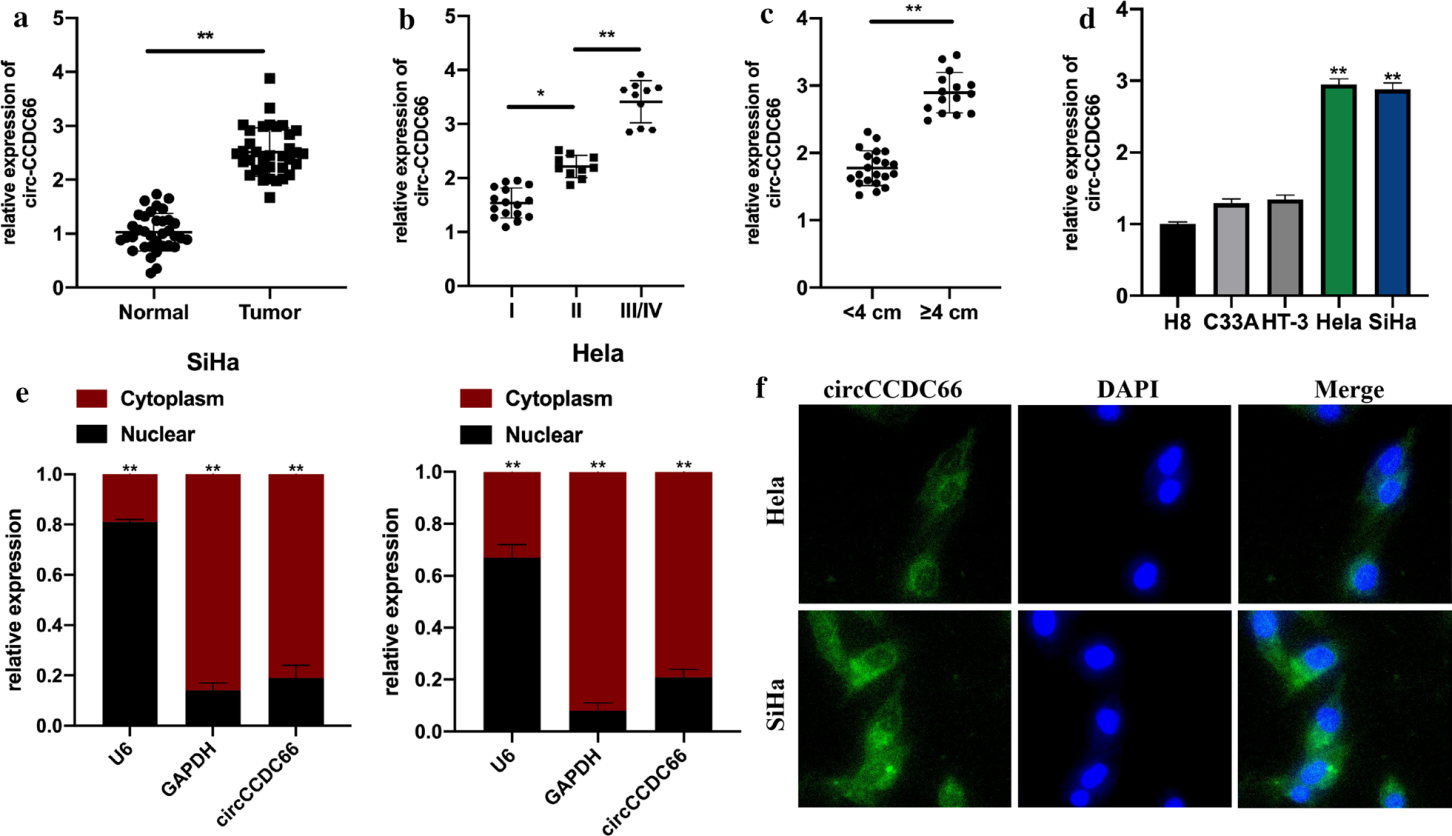
Circ-CCDC66 is upregulated in cervical cancer tissues. **a** The expression of circ-CCDC66 in 36 pairs of cervical cancer tissues and its matched normal tissues were analyzed by qRT-PCR. **b** and **c** relative expression of CCDC66 was analyzed according to TNM stage and tumor size using qRT-PCR. **d** relative expression of circ-CCDC66 in cervical cancer cell lines HeLa, SiHa, C33A, HT-3, and normal cervical cell line H8 were assessed by qRT-PCR. **e** nuclear-cytoplasmic fractionation assays were conducted to detect the expression of CCDC66 in the nuclear or cytoplasmic of cervical cells. **f** The location of CCDC66 in cervical cells were detected by FISH assay. Data were presented as mean ± SD, all experiments were repeated at least three times. Scare bar for FISH image: 20 μm. *P < 0.05, **P < 0.01

The expression of circ-CCDC66 in 36 pairs of cervical cancer human samples were measured. Circ-CCDC66 was significantly upregulated in tumor tissues compared with matched normal tissues (Fig. 1a).High expression of circ-CCDC-66 was found to associate with large tumor size and advanced tumor stage (Fig. 1b-c). Our findings indicated that circ-CCDC66 might be involved in the progression of cervical cancer. Subsequently, it was found that circ-CCDC66 was upregulated in cervical cancer cell lines compared with normal cervical cell line H8, especially in Hela and SiHa cells (Fig. 1d), and mainly located in cell cytoplasm (Fig. 1e and f).

### Circ-CCDC66 promotes the proliferation, migration and invasion of cervical cancer cells

**Fig. 2:**
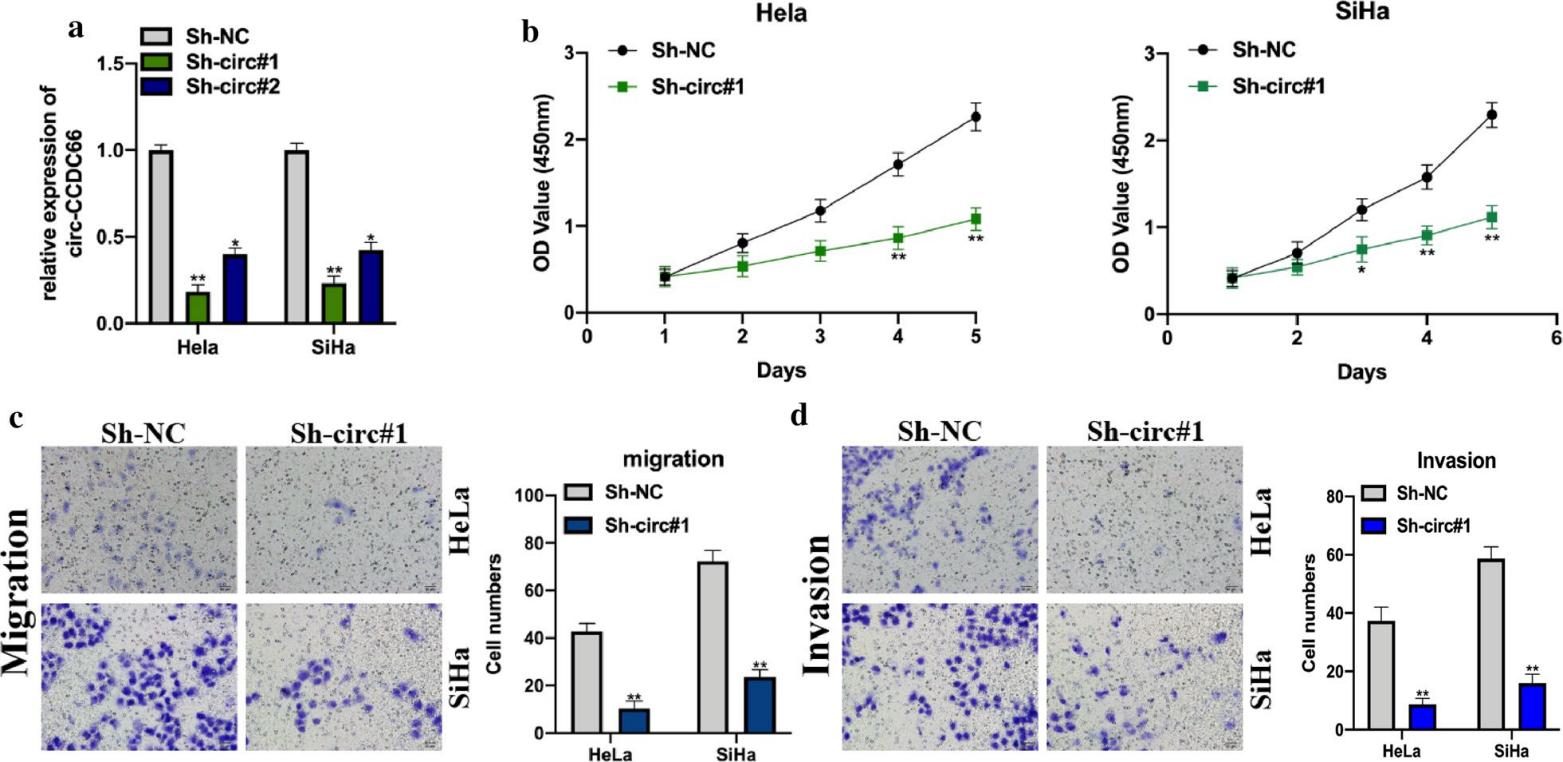
Circ-CCDC66 promotes the proliferation, migration and invasion of cervical cancer cells. Cervical cancer cell lines HeLa and SiHa were stably transfected with Sh-NC or Sh-circ-CCDC66#1, Sh-circ-CCDC66#2 to generate circ-CCDC66 knockdown cell models. **a** relative expression of circ-CCDC66 in treated HeLa and SiHa cells were analyzed by qRT-PCR assay. **b** Cell proliferation abilities of treated HeLa and SiHa cells were detected by CCK-8 assays. **c** Transwell migration assays were conducted to evaluate cell migration ability. **d** Transwell invasion assays were performed to measure cell invasion ability. Data were presented as mean ± SD, all experiments were repeated at least three times. Scare bar for Transwell image: 100 μm *P < 0.05, **P < 0.01

To elucidate the biological function of circ-CCDC66, we constructed circ-CCDC66 knockdown cell models by stably transfecting Sh-NC or Sh-circ-CCDC66 into Hela and SiHa cells, the transfection efficiencies were detected (Fig. 2a). By performing CCK-8 and transwell assays, it was found that circ-CCDC66 knockdown significantly suppressed cervical cancer cell proliferation, migration, and invasion abilities (Fig. 2b-d). Furthermore, overexpression of circ-CCDC66 in cervical cancer cells were couple with promotive phenomenon in cell proliferation, migration and invasion (Additional file 1: Figure S1). These results suggested that circ-CCDC66 regulated cervical cancer cellular progression.

### Circ-CCDC66 acts as an efficient molecular sponge for miR-452-5p

**Fig. 3:**
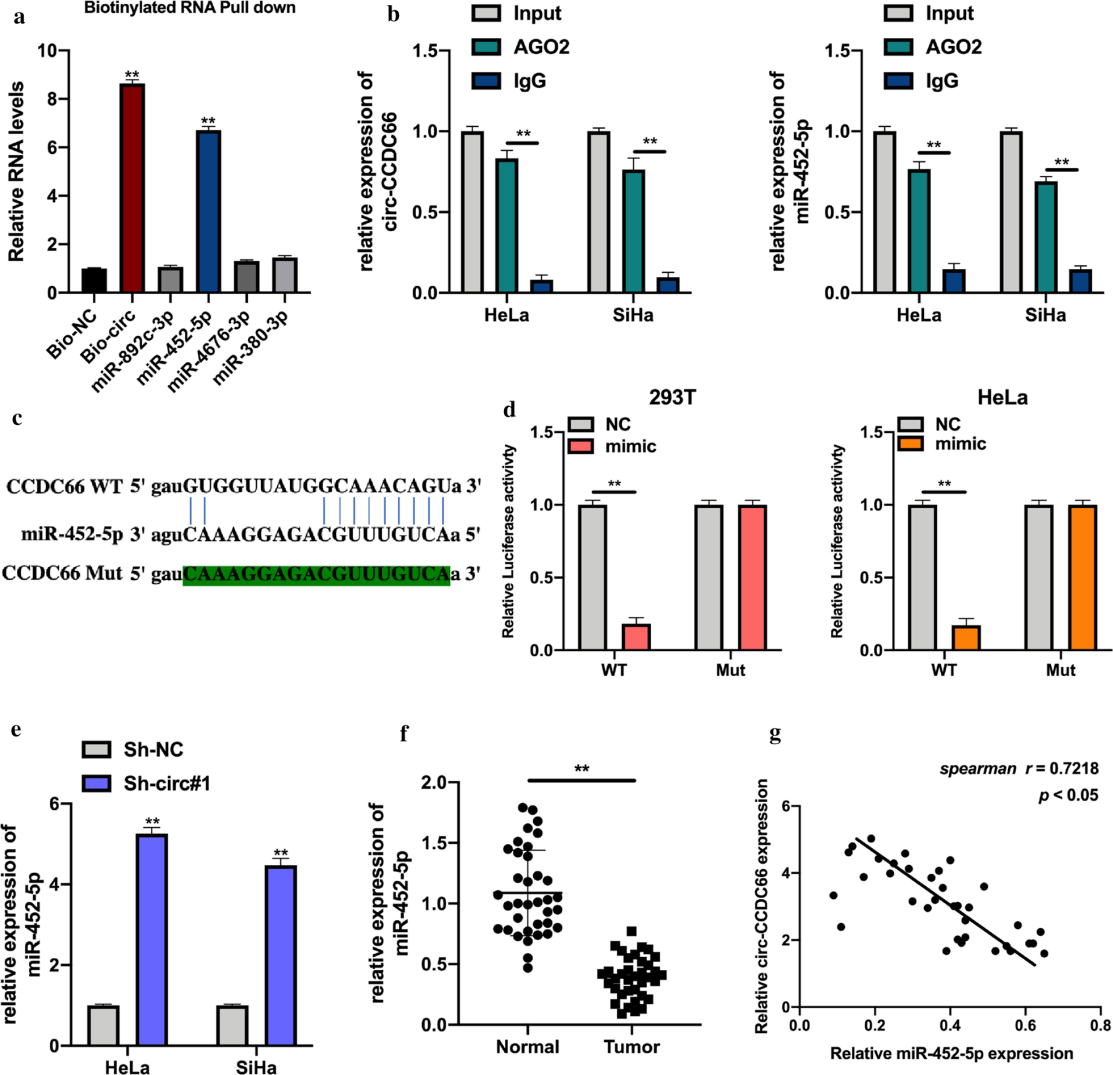
Circ-CCDC66 acts as a molecular sponge for miR-452-5p. Putative downstream miRNAs of circ-CCDC66 were predicted by Miranda (http://miranda.org.uk/) database, four miRNAs were selected for our further study. **a** Full length of circ-CCDC66 were biotinylated, and the complexes were subjected to qRT-PCR assays. Relative miRNAs expression were presented. **b** AGO-2 RIP assays were conducted, and the expression of circ-CCDC66 and miR-452-5p in protein bonds were detected by qRT-PCR. **c** The binding sites between circ-CCDC66 and miR-452-5p were showed. **d** Luciferase reporter assays were used to determine the interaction between circ-CCDC66 and miR-452-5p in 293 T and Hela cells. **e** The expression of miR-452-5p in Sh-NC or Sh-circCCDC66#1 transfected Hela and SiHa cells were detected by qRT-PCR. **f** the expression of miR-452-5p in cervical cancer tissues were measured by qRT-PCR. **g **Spearman analysis was employed to assess the correlation expression between circ-CCDC66 and miR-452-5p. Data were presented as mean ± SD, all experiments were performed at least three times. **P < 0.01

Previous studies demonstrated that circRNA plays its role via sponging to miRNAs and mediating downstream gene expression [23, 24]. In the current study, the downstream factor of circ-CCDC66 was predicted using Miranda database. Four miRNAs were selected (CLIP data: strict stringency (≥5)) for investigation. It was found that miR-452-5p was highly enriched in biotinylated circ-CCDC66 probe bounds (Fig. 3a, Additional file 1: Figure S3), and both circ-CCDC66 and miR-452-5p were enriched in anti-AGO2 complexes (Fig. 3b), suggesting that circ-CCDC66 might interact with miR-452-5p. The wild type (WT) and mutant type (Mut) sequence of circ-CCDC66 binding with miR-452-5p were synthesized (Fig. 3c). The luciferase activities in vectors containing circ-CCDC66 WT sequence and miR-452-5p mimics co-transfected 293 T and Hela cells were significantly decreased (Fig. 3d). Subsequently, it was found that circ-CCDC66 negatively regulated miR-452-5p expression (Fig. 3e). Furthermore, our results found that miR-452-5p was downregulated in cervical cancer tumor tissues compared with matched normal tissues, and statistically correlated with circ-CCDC66 expression (Fig. 3f and g).

### REXO1 is a downstream target for miR-452-5p

**Fig. 4:**
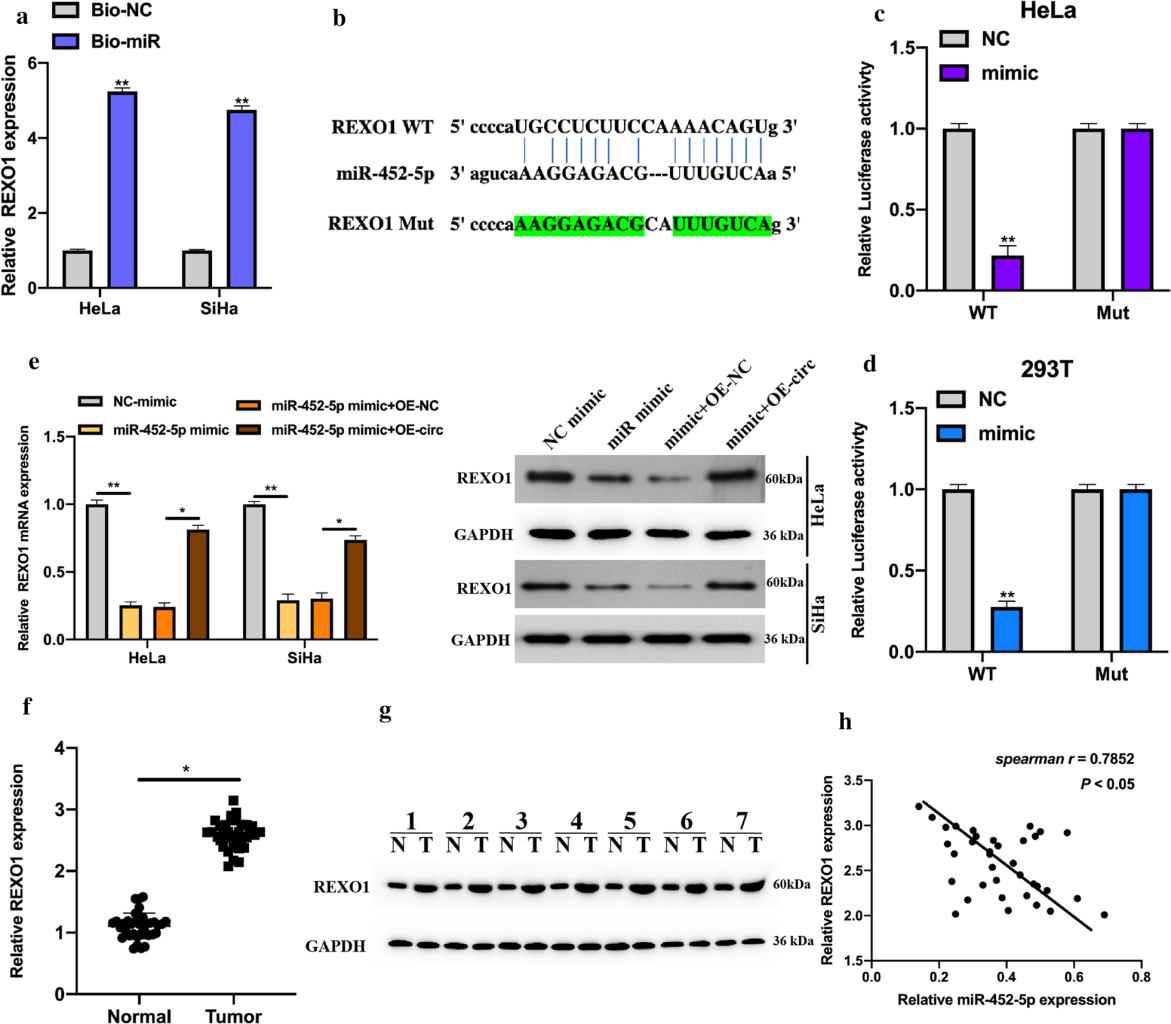
REXO1 is a downstream target for miR-452-5p. Potential mRNA targets of miR-452-5p were predicted by PITA (https://genie.weizmann.ac.il/pubs/mir07/mir07_exe.html), RNA22V2 (https://cm.jefferson.edu/rna22/), miRmap (https://mirmap.ezlab.org/downloads/mirmap201301e/), TargetScan Human 7.2 (http://www.targetscan.org/vert_72/) dataset. **a** Bio-miR-452-5p and Bio-NC probes were constructed, and qRT-PCR was applied to analyze the expression of REXO1 mRNA in the purified bonds. **b** the binding sites between miR-452-5p and REXO1, mutant sequence of REXO1 was synthesized. **c** and **d** Luciferase reporter assays were performed to determine the correlation between miR-452-5p and REXO1 in 293 T and Hela cells. **e** Hela and SiHa cells were stably transfected with NC mimic, miR-452-5p mimic, miR-452-5p mimic with OE-NC, and miR-452-5p mimic with OE-circ-CCDC66 as indicated, the protein and mRNA levels of REXO1 were measured by qRT-PCR and western blot. **f** The expression of REXO1 mRNA in cervical cancer tissues were evaluated by qRT-PCR. **g** The protein expression in randomly selected seven pairs of cervical cancer human samples were detected by western blot. **h** The expression of miR-452-5p and REXO1 in tumor tissues were analyzed by spearman analysis. Data were presented as mean ± SD, all experiments were performed at least three times. *P < 0.05, **P < 0.01

Downstream target of miR-452-5p was investigated. Firstly, we found that REXO1 mRNA was abundantly enriched in bio-miR-452-5p probe bounds (Fig. 4a), and the binding sites between miR-452-5p and REXO1 were synthesized (Fig. 4b). Next, luciferase activities in vectors harboring REXO1 WT sequences and miR-452-5p mimics co-transfected 293 T and HeLa cells were significantly decreased (Fig. 4 c and d). Furthermore, the expression of REXO1 was suppressed by miR-452-5p mimic, but rescued by OE-circ-CCDC66 (Fig. 4e). Moreover, REXO1 was found to highly express in cervical cancer tumor tissues, and strongly correlate with miR-452-5p expression (Fig. 4 f–h).

### Circ-CCDC66 mediates REXO1 expression to promote cervical cancer progression via miR-452-5p

**Fig. 5:**
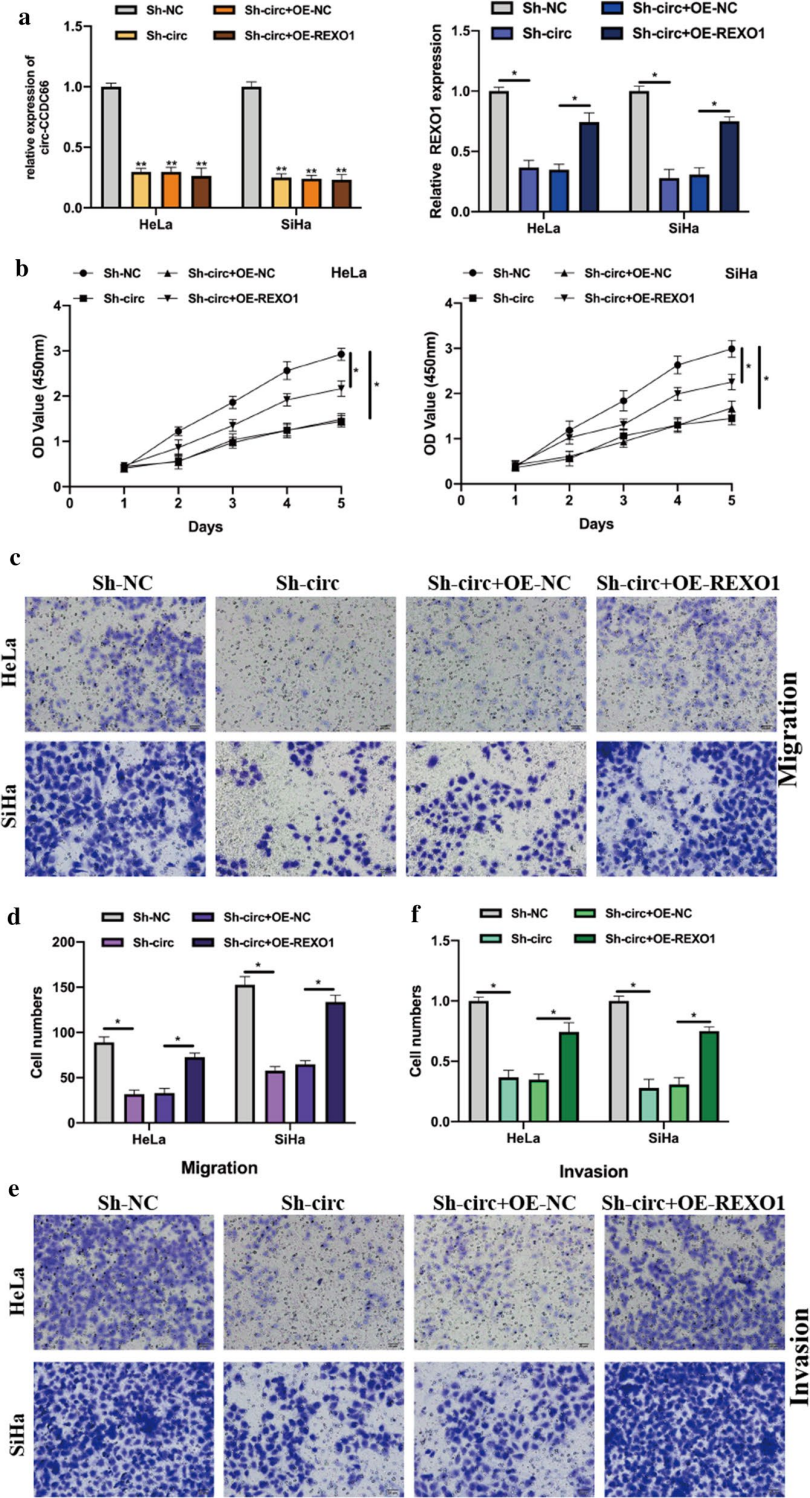
Circ-CCDC66 mediates REXO1 expression to promote cervical cancer progression via miR-452-5p. Cell models were constructed by transfecting Sh-NC, Sh-circ-CCDC66, Sh-circ-CCDC66 + OE-NC, Sh-circ-CCDC66 + OE-REXO1 into Hela and SiHa cells. **a** transfection efficiencies were detected by qRT-PCR. **b** Cells were subjected to CCK-8 assays for cell proliferation detection. **c** and **d** Transwell migration assays were performed to evaluate cell migration abilities, number of migrated cells were recorded. **e** and **f** Cell invasion abilities were determined by transwell invasion assays, invasion cells were recorded and comparative statistics were presented. Data were presented as mean ± SD, all experiments were performed at least three times. Scare bar for Transwell image: 100 μm *P < 0.05, **P < 0.01

The biological effects of REXO1 on cervical cancer cells were evaluated. It was found that over expression of REXO1 promoted cell proliferation, migration, and invasion (Additional file 1: Figure S2). Subsequently, we investigated the role of circ-CCDC66/miR-452-5p/REXO1 axis in cervical cancer cellular progression. Cell models were generated by transfecting Sh-NC, Sh-circ-CCDC66, Sh-circ-CCDC66 + OE-NC, Sh-circ-CCDC66 + OE-REXO1 into Hela and SiHa cells. The expression of cric-CCDC66 and REXO1 were measured (Fig. 5a). CCK-8 and Transwell assays showed that the inhibitory effects of downregulated circ-CCDC66 on Hela and SiHa cells were rescued by REXO1 overexpression (Fig. 5 b-f), suggested that circ-CCDC66 regulated REXO1 expression to promote cervical cancer progression via sponging miR-452-3p.

### Downregulation of circ-CCDC66 inhibits cervical cancer cell growth in vivo.

**Fig. 6:**
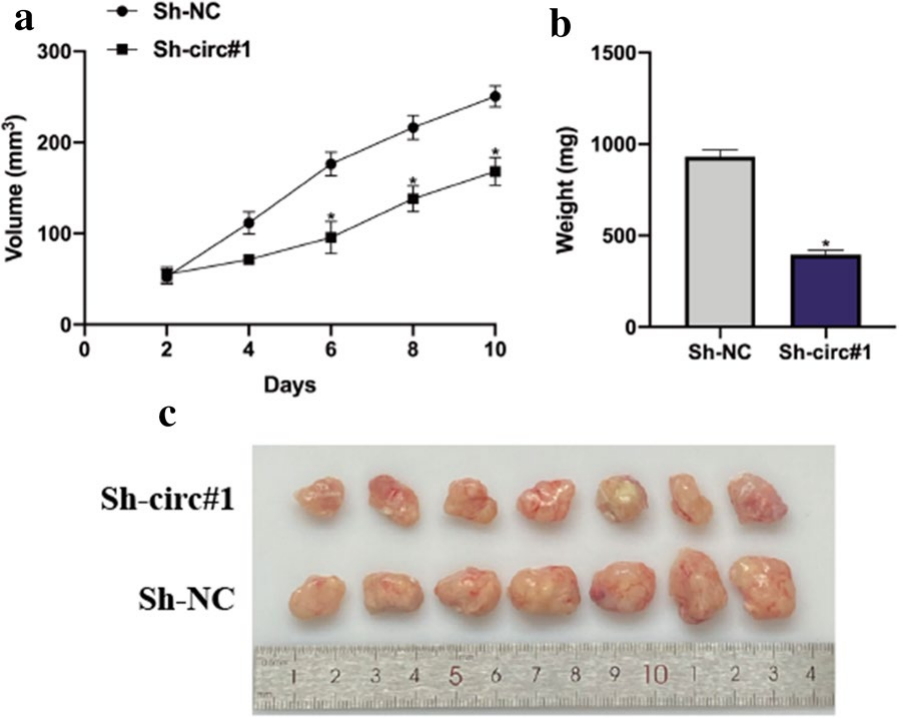
Downregulation of circ-CCDC66 inhibits cervical cancer cell growth in vivo. Hela cell (10^6^ per tumor) pre-transfected with Sh-NC or Sh-circCCDC66 were subcutaneously injected into node mouse (7 mice for each group). After 28 days, tumors were harvested after mice were sacrificed. **a** and **b** tumor volumes and tumor end weights were recorded. **c** Representative image of xenotransplantation tumors were showed. Data were presented as mean ± SD, all experiments were performed at least three times. *P < 0.05

The function of circ-CCDC66 in vivo was investigated through constructing circ-CCDC66 knockdown mouse models. As shown in Fig. 6 a and b, circ-CCDC66 knockdown significantly suppressed cervical cancer cell growth in vivo, the representative image of xenotransplantation tumors showed the inhibitory effect of circ-CCDC66 (Fig. 6c).

## Discussion

Cervical cancer has been identified as a gynecologic malignancy cancer type with high morbidity and mortality rate in various cancers, and presents great health threat to female worldwide [25, 26]. Due to its inefficiency in diagnosis and clinical intervention, finding novel therapeutic targets for cervical cancer treatment become urgent.

In this study, we revealed that circ-CCDC66 acted as an oncogene in cervical cancer progression. Firstly, circ-CCDC66 expression in cervical cancer human samples were assessed, our findings suggested that circ-CCDC66 was highly expressed in tumor tissues, and associated with later tumor stage and lager tumor size, indicated that circ-CCDC66 might be involved in cervical cancer progression. Previous study has demonstrated that circ-CCDC66 mediates tumorigenesis via regulating multiple cellular functions, such as proliferation, migration, invasion and EMT levels [18, 19, 27], suggested the important role of circ-CCDC66 in tumorigenesis progression. Here, knockdown of circ-CCDC66 was found to attenuate cervical cancer cell proliferation, migration and invasion abilities in vitro, and inhibit cervical cancer cell growth in vivo.

CircRNAs have been widely reported that mediate virous biological progressions through transcriptionally regulating gene expression via sponging microRNA [23, 24, 28, 29]. To investigate the underlying molecular mechanisms of circ-CCDC66 in cervical cancer progression. Bioinformatics analysis, RNA-pull down, RIP, and dual-luciferase reporter assays was conducted. It was found that circ-CCDC66 was an efficient molecular sponge for miR-452-5p, and miR-452-5p directly targeted to REXO1 in cervical cancer cells. MiR-452-5p has been revealed that participates in multiple cancer progression, such as: renal cancer, prostate cancer, hepatocellular caner, and lung squamous cell carcinoma [30–33], and showed its specific role in tumorigenesis. While, the function of miR-452-5p in cervical cancer remains unelucidated. Our study found that circ-CCDC66 sponged to miR-452-5p and negatively regulated its expression in cervical cancer cells. Furthermore, it was found that miR-452-5p was downregulated in cervical cancer tumor tissues in comparison with matched normal tissues. Subsequently, our study found that miR-452-5p directly targeted to REXO1, and negatively regulated REXO1 expression in cervical cancer cells. CCK-8 and Transwell assays’ results showed that overexpression of REXO1 reversed the inhibitory effects of circ-CCDC66 on cell proliferation, migration, invasion abilities.

Despite the role of circ-CCDC66/miR-452-5p/REXO1 pathway has been partially revealed by our study. On the one hand, our results were limited by small human sample size. One the other hand, the downstream molecular mechanisms of REXO1 in cervical cancer progression need further exploration.

## Conclusions

Our study revealed that the expression of circ-CCDC66 was upregulated in cervical cancer tumor tissues, and circ-CCDC66 mediated the proliferation, migration, and invasion abilities of cervical cancer cells. Moreover, our results demonstrated that circ-CCDC66 promoted cervical cancer progression via miR-452-5p/REXO1 axis, indicating that circ-CCDC66 might be a novel therapeutic target for cervical cancer.

## Supplementary Information

**Additional file 1**: **Figure S1.** Circ-CCDC66 promotes the proliferation, migration and invasion of cervical cancer cells. A: Cervical cancer cell HeLa and SiHa were transfected with OE-NC or OE-circ-CCDC66, transfection efficiencies were assessed by qRT-PCR. B: Cell proliferation abilities of treated HeLa and SiHa were detected by CCK-8 assays. C: Transwell migration assays were conducted to evaluate cell migration ability. D: Transwell invasion assays were performed to measure cell invasion ability. Data were presented as mean ± SD, all experiments were repeated at least three times. *P < 0.05, **P < 0.01. **Figure S2.** REXO1 overexpression promotes cervical cancer cell proliferation, migration and invasion. A: Cervical cancer cell HeLa and SiHa were transfected with OE-NC or OE-REXO1, qRT-PCR was conducted to evaluate transfection efficiencies. B: Cell proliferation abilities were measured by CCK-8 assays. C: Transwell migration assays were conducted to evaluate cell migration ability. D: Transwell invasion assays were performed to measure cell invasion ability. Data were presented as mean ± SD, all experiments were repeated at least three times. *P < 0.05, **P < 0.01. **Figure S3.** The blots for biotinylated RNA pull down of circ-CCDC66. Circ-CCDC66 in the HeLa and SiHa lysates was pulled down and enriched with circ-CCDC66 specific probe and then detected by qRT-PCR. Relative level of circ-CCDC66 was normalized to the input. GAPDH was used as a negative control.

## Data Availability

The data performed and analyzed during the present study are available from the corresponding author on reasonable request.

## References

[1] Torre LA, Bray F, Siegel RL, Ferlay J, Lortet-Tieulent J, Jemal A (2015). Global cancer statistics, 2012. CA Cancer J Clin.

[2] Zheng RS, Sun KX, Zhang SW, Zeng HM, Zou XN, Chen R, Gu XY, Wei WW, He J (2019). Report of cancer epidemiology in China, 2015. Zhonghua Zhong Liu Za Zhi.

[3] Feng LL, Shen FR, Zhou JH, Chen YG (2019). Expression of the lncRNA ZFAS1 in cervical cancer and its correlation with prognosis and chemosensitivity. Gene.

[4] Origoni M, Prendiville W, Paraskevaidis E (2015). Cervical cancer prevention: new frontiers of diagnostic strategies. Biomed Res Int.

[5] Barrett SP, Wang PL, Salzman J (2015). Circular RNA biogenesis can proceed through an exon-containing lariat precursor. Elife.

[6] Jeck WR, Sorrentino JA, Wang K, Slevin MK, Burd CE, Liu J, Marzluff WF, Sharpless NE (2013). Circular RNAs are abundant, conserved, and associated with ALU repeats. RNA.

[7] Suzuki H, Tsukahara T (2014). A view of pre-mRNA splicing from RNase R resistant RNAs. Int J Mol Sci.

[8] Di Agostino S, Riccioli A, De Cesaris P, Fontemaggi G, Blandino G, Filippini A, Fazi F (2020). Circular RNAs in embryogenesis and cell differentiation with a focus on cancer development. Front Cell Dev Biol.

[9] Song H, Liu Q, Liao Q (2020). Circular RNA and tumor microenvironment. Cancer Cell Int.

[10] Chen LL, Yang L (2015). Regulation of circRNA biogenesis. RNA Biol.

[11] Chaichian S, Shafabakhsh R, Mirhashemi SM, Moazzami B, Asemi Z (2020). Circular RNAs: A novel biomarker for cervical cancer. J Cell Physiol.

[12] Gao YL, Zhang MY, Xu B, Han LJ, Lan SF, Chen J, Dong YJ, Cao LL (2017). Circular RNA expression profiles reveal that hsa_circ_0018289 is up-regulated in cervical cancer and promotes the tumorigenesis. Oncotarget.

[13] Song T, Xu A, Zhang Z, Gao F, Zhao L, Chen X, Gao J, Kong X (2019). CircRNA hsa_circRNA_101996 increases cervical cancer proliferation and invasion through activating TPX2 expression by restraining miR-8075. J Cell Physiol.

[14] Tang Q, Chen Z, Zhao L, Xu H (2019). Circular RNA hsa_circ_0000515 acts as a miR-326 sponge to promote cervical cancer progression through up-regulation of ELK1. Aging (Albany NY).

[15] Glazar P, Papavasileiou P, Rajewsky N (2014). circBase: a database for circular RNAs. RNA.

[16] Yang R, Wang Z, Meng G, Hua L (2020). Circular RNA CCDC66 facilitates abdominal aortic aneurysm through the overexpression of CCDC66. Cell Biochem Funct..

[17] Hsiao KY, Lin YC, Gupta SK, Chang N, Yen L, Sun HS, Tsai SJ (2017). Noncoding effects of circular RNA CCDC66 promote colon cancer growth and metastasis. Cancer Res.

[18] Yang M, Wang GY, Qian H, Ji XY, Liu CY, Zeng XH, Lv J, Shi YX (2019). Circ-CCDC66 accelerates proliferation and invasion of gastric cancer via binding to miRNA-1238-3p. Eur Rev Med Pharmacol Sci.

[19] Wen Z, Shen Q, Zhang H, Su Y, Zhu Z, Chen G, Peng L, Li H, Du C, Xie H, Xu X, Tang W (2019). Circular RNA CCDC66 targets DCX to regulate cell proliferation and migration by sponging miR-488-3p in Hirschsprung's disease. J Cell Physiol.

[20] Cai H, Zhang P, Xu M, Yan L, Liu N, Wu X (2019). Circular RNA hsa_circ_0000263 participates in cervical cancer development by regulating target gene of miR-150-5p. J Cell Physiol.

[21] Li Y, Zheng F, Xiao X, Xie F, Tao D, Huang C, Liu D, Wang M, Wang L, Zeng F, Jiang G (2017). CircHIPK3 sponges miR-558 to suppress heparanase expression in bladder cancer cells. EMBO Rep.

[22] Wang K, Long B, Liu F, Wang JX, Liu CY, Zhao B, Zhou LY, Sun T, Wang M, Yu T, Gong Y, Liu J, Dong YH, Li N, Li PF (2016). A circular RNA protects the heart from pathological hypertrophy and heart failure by targeting miR-223. Eur Heart J.

[23] Kristensen LS, Andersen MS, Stagsted LVW, Ebbesen KK, Hansen TB, Kjems J (2019). The biogenesis, biology and characterization of circular RNAs. Nat Rev Genet.

[24] Patop IL, Wust S, Kadener S (2019). Past, present, and future of circRNAs. EMBO J.

[25] Global Burden of Disease Cancer C, Fitzmaurice C, Dicker D, Pain A, Hamavid H, Moradi-Lakeh M, MacIntyre MF, Allen C, Hansen G, Woodbrook R, Wolfe C, Hamadeh RR, Moore A, Werdecker A, Gessner BD, Te Ao B, McMahon B, Karimkhani C, Yu C, Cooke GS, Schwebel DC, Carpenter DO, Pereira DM, Nash D, Kazi DS, De Leo D, Plass D, Ukwaja KN, Thurston GD, Yun Jin K, Simard EP, Mills E, Park EK, Catala-Lopez F, deVeber G, Gotay C, Khan G, Hosgood HD, 3rd, Santos IS, Leasher JL, Singh J, Leigh J, Jonas JB, Sanabria J, Beardsley J, Jacobsen KH, Takahashi K, Franklin RC, Ronfani L, Montico M, Naldi L, Tonelli M, Geleijnse J, Petzold M, Shrime MG, Younis M, Yonemoto N, Breitborde N, Yip P, Pourmalek F, Lotufo PA, Esteghamati A, Hankey GJ, Ali R, Lunevicius R, Malekzadeh R, Dellavalle R, Weintraub R, Lucas R, Hay R, Rojas-Rueda D, Westerman R, Sepanlou SG, Nolte S, Patten S, Weichenthal S, Abera SF, Fereshtehnejad SM, Shiue I, Driscoll T, Vasankari T, Alsharif U, Rahimi-Movaghar V, Vlassov VV, Marcenes WS, Mekonnen W, Melaku YA, Yano Y, Artaman A, Campos I, MacLachlan J, Mueller U, Kim D, Trillini M, Eshrati B, Williams HC, Shibuya K, Dandona R, Murthy K, Cowie B, Amare AT, Antonio CA, Castaneda-Orjuela C, van Gool CH, Violante F, Oh IH, Deribe K, Soreide K, Knibbs L, Kereselidze M, Green M, Cardenas R, Roy N, Tillmann T, Li Y, Krueger H, Monasta L, Dey S, Sheikhbahaei S, Hafezi-Nejad N, Kumar GA, Sreeramareddy CT, Dandona L, Wang H, Vollset SE, Mokdad A, Salomon JA, Lozano R, Vos T, Forouzanfar M, Lopez A, Murray C, Naghavi M. The Global Burden of Cancer 2013. *JAMA Oncol*. 2015; 1: 505–27. 10.1001/jamaoncol.2015.0735 PMC4500822 26181261

[26] Tewari KS, Sill MW, Long HJ, Penson RT, Huang H, Ramondetta LM, Landrum LM, Oaknin A, Reid TJ, Leitao MM, Michael HE, Monk BJ (2014). Improved survival with bevacizumab in advanced cervical cancer. N Engl J Med.

[27] Joseph NA, Chiou SH, Lung Z, Yang CL, Lin TY, Chang HW, Sun HS, Gupta SK, Yen L, Wang SD, Chow KC (2018). The role of HGF-MET pathway and CCDC66 cirRNA expression in EGFR resistance and epithelial-to-mesenchymal transition of lung adenocarcinoma cells. J Hematol Oncol.

[28] Panda AC (2018). Circular RNAs act as miRNA sponges. Adv Exp Med Biol.

[29] Li X, Yang L, Chen LL (2018). The biogenesis, functions, and challenges of circular RNAs. Mol Cell.

[30] Gan XN, Gan TQ, He RQ, Luo J, Tang RX, Wang HL, Zhou H, Qing H, Ma J, Hu XH, Chen G (2018). Clinical significance of high expression of miR-452-5p in lung squamous cell carcinoma. Oncol Lett.

[31] Rong MH, Cai KT, Lu HP, Guo YN, Huang XY, Zhu ZH, Tang W, Huang SN (2019). Overexpression of MiR-452-5p in hepatocellular carcinoma tissues and its prospective signaling pathways. Int J Clin Exp Pathol.

[32] Gao L, Zhang LJ, Li SH, Wei LL, Luo B, He RQ, Xia S (2018). Role of miR-452-5p in the tumorigenesis of prostate cancer: a study based on the Cancer Genome Atl(TCGA), Gene Expression Omnibus (GEO), and bioinformatics analysis. Pathol Res Pract.

[33] Zhai W, Li S, Zhang J, Chen Y, Ma J, Kong W, Gong D, Zheng J, Xue W, Xu Y (2018). Sunitinib-suppressed miR-452-5p facilitates renal cancer cell invasion and metastasis through modulating SMAD4/SMAD7 signals. Mol Cancer.

