# Supplementary material for: Circ-CCDC66 upregulates REXO1 expression to aggravate cervical cancer progression via restraining miR-452-5p
Source: Cancer Cell Int. 2021 Jan 6;21:20. doi: 10.1186/s12935-020-01732-8 (PMC7789749; doi:10.1186/s12935-020-01732-8)
Supplement: Supplementary file 1 — Additional file 1: Figure S1. Circ-CCDC66 promotes the proliferation, migration and invasion of cervical cancer cells. A: Cervical cancer cell HeLa and SiHa were transfected with OE-NC or OE-circ-CCDC66, transfection efficiencies were assessed by qRT-PCR. B: Cell proliferation abilities of treated HeLa and SiHa were detected by CCK-8 assays. C: Transwell migration assays were conducted to evaluate cell migration ability. D: Transwell invasion assays were performed to measure cell invasion ability. Data were presented as mean ± SD, all experiments were repeated at least three times. *P < 0.05, **P < 0.01. Figure S2. REXO1 overexpression promotes cervical cancer cell proliferation, migration and invasion. A: Cervical cancer cell HeLa and SiHa were transfected with OE-NC or OE-REXO1, qRT-PCR was conducted to evaluate transfection efficiencies. B: Cell proliferation abilities were measured by CCK-8 assays. C: Transwell migration assays were conducted to evaluate cell migration ability. D: Transwell invasion assays were performed to measure cell invasion ability. Data were presented as mean ± SD, all experiments were repeated at least three times. *P < 0.05, **P < 0.01. Figure S3. The blots for biotinylated RNA pull down of circ-CCDC66. Circ-CCDC66 in the HeLa and SiHa lysates was pulled down and enriched with circ-CCDC66 specific probe and then detected by qRT-PCR. Relative level of circ-CCDC66 was normalized to the input. GAPDH was used as a negative control. [file 12935_2020_1732_MOESM1_ESM.docx]

**This file includes:**

**Additional Figures S1-S3**

**
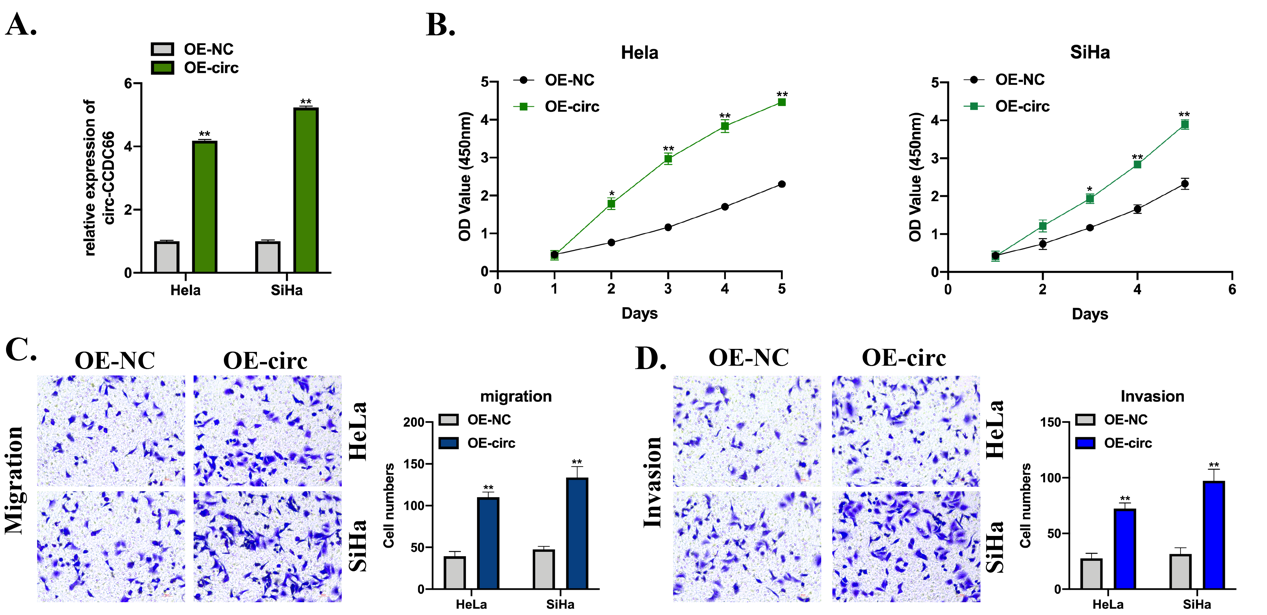
**

**Figure S1. Circ-CCDC66 promotes the proliferation, migration and invasion of cervical cancer cells. A**: Cervical cancer cell HeLa and SiHa were transfected with OE-NC or OE-circ-CCDC66, transfection efficiencies were assessed by qRT-PCR. B: Cell proliferation abilities of treated HeLa and SiHa were detected by CCK-8 assays. C: Transwell migration assays were conducted to evaluate cell migration ability. D: Transwell invasion assays were performed to measure cell invasion ability. Data were presented as mean ± SD, all experiments were repeated at least three times. **P* < 0.05, ***P* < 0.01.


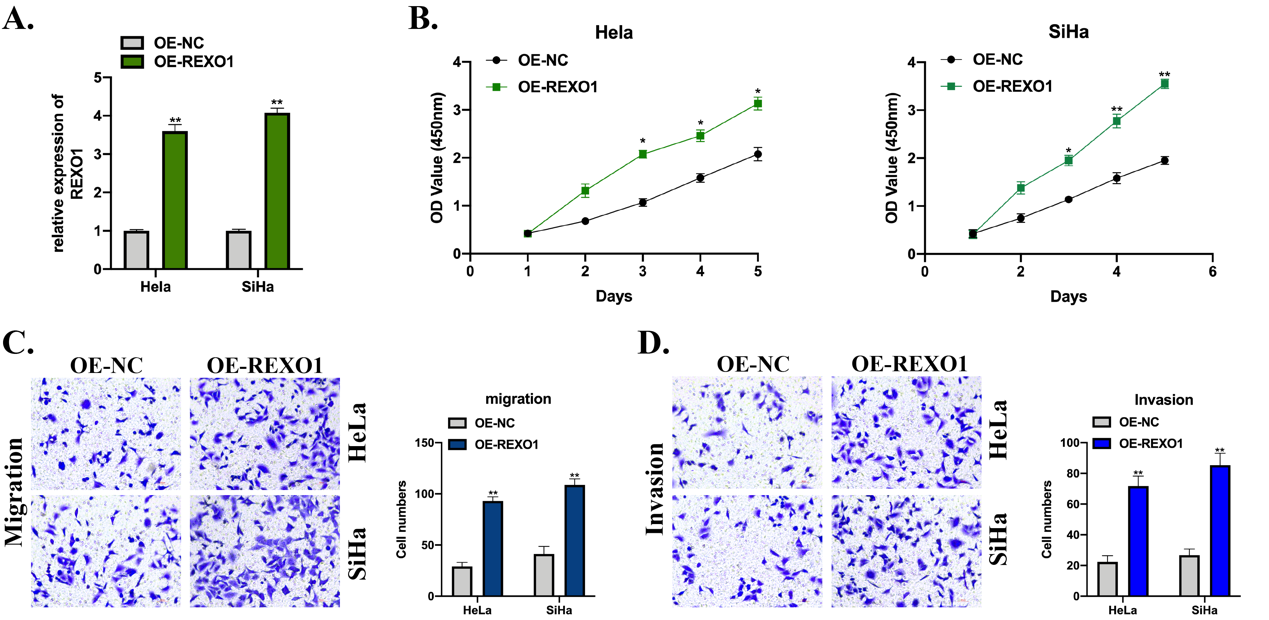


**Figure S2. REXO1 overexpression promotes cervical cancer cell proliferation, migration and invasion. A**: Cervical cancer cell HeLa and SiHa were transfected with OE-NC or OE-REXO1, qRT-PCR was conducted to evaluate transfection efficiencies. B: Cell proliferation abilities were measured by CCK-8 assays. C: Transwell migration assays were conducted to evaluate cell migration ability. D: Transwell invasion assays were performed to measure cell invasion ability. Data were presented as mean ± SD, all experiments were repeated at least three times. **P* < 0.05, ***P* < 0.01.

**
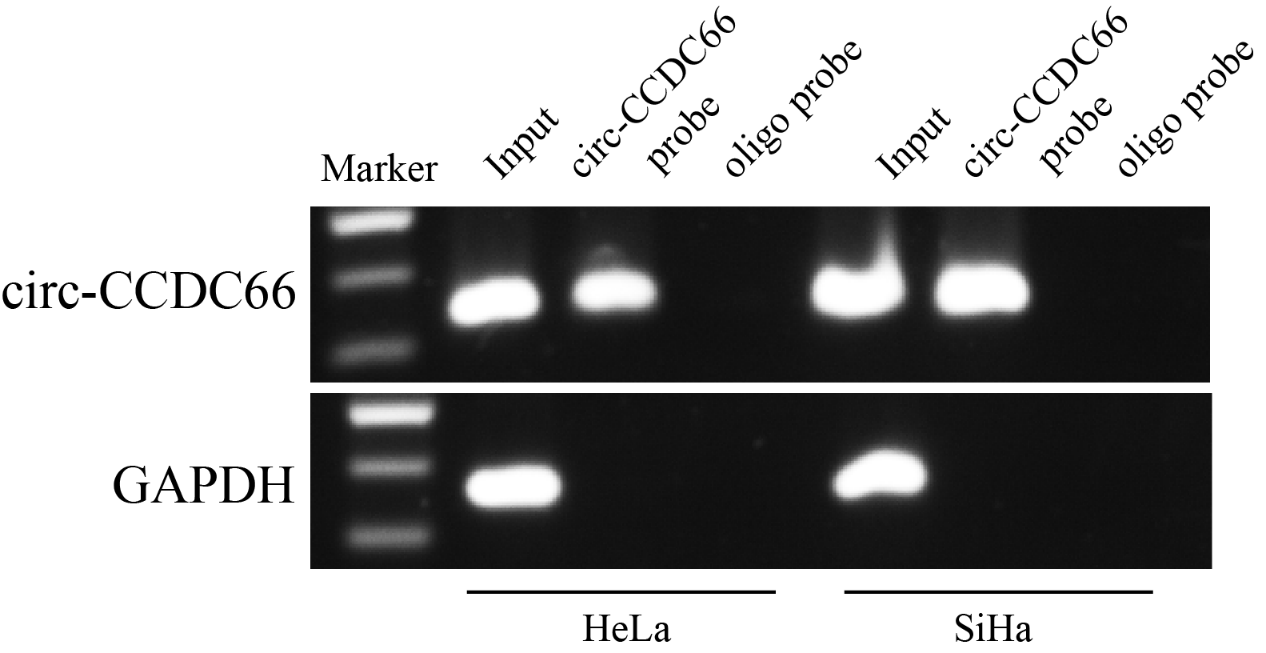
**

**Figure S3. The blots for biotinylated RNA pull down of circ-CCDC66.** Circ-CCDC66 in the HeLa and SiHa lysates was pulled down and enriched with circ-CCDC66 specific probe and then detected by qRT-PCR. Relative level of circ-CCDC66 was normalized to the input. GAPDH was used as a negative control.
